# A Rare Case of a Dentinogenic Ghost Cell Tumor Associated With a Compound Odontoma: A Case Report and Literature Review

**DOI:** 10.7759/cureus.69496

**Published:** 2024-09-16

**Authors:** Madhusudhan Reddy, Kishore Moturi, Vadde Venkata Naga Sunil, Mrunalini Koneru, G Pradeep Raj

**Affiliations:** 1 Oral Pathology and Microbiology, Army College of Dental Sciences, Secunderabad, IND; 2 Oral and Maxillofacial Surgery, Army College of Dental Sciences, Secunderabad, IND; 3 General Dentistry, Army College of Dental Sciences, Secunderabad, IND; 4 Orthodontics and Dentofacial Orthopaedics, Army College of Dental Sciences, Secunderabad, IND

**Keywords:** compound odontome, dentinogenic ghost cell tumor, ghost cells, mixed epithelial mesenchymal odontogenic tumor, odontogenic tumor

## Abstract

A dentinogenic ghost cell tumor (DGCT) is a rare and locally aggressive benign mixed odontogenic tumor, histologically made up of ameloblast-like epithelial islands, ghost cells, and dentin-like material. This is a highly unusual example of DGCT combined with an odontoma in a 13-year-old female patient affecting the right maxilla. On radiographic examination, ill-defined radiolucency with right maxillary sinus obliteration and involvement of permanent maxillary right first and second molars were noted. The second molar was pushed towards the orbital fossa, and root resorption of the first molar is associated with multiple radiopaque masses. The excision specimen showed multiple tooth-like structures histologically, including dentin-like areas and dental pulp. Only five cases of a DGCT with an odontoma have been documented in the literature, making it a very unusual condition.

## Introduction

Dentinogenic ghost cell tumors (DGCTs) which are benign mixed odontogenic tumors are less than 0.5% of all odontogenic tumors. These are rare and arise from the remaining dental lamina or enamel epithelium. The term was first introduced in 1981 by Praetorius et al.. It is considered a separate entity in WHO 2005 classification and has maintained its entity status without any important updates in WHO 2017 and 2022 classifications [1-4].

According to the WHO classification, the dualistic concept of calcifying odontogenic cysts (COCs) has two entities. The cystic entity is termed calcifying cystic odontogenic tumor (CCOT), and DGCT is the word for a neoplastic entity. It is characterized as a “locally invasive neoplasm characterized by ameloblastoma-like islands of epithelial cells in a mature connective tissue stroma. Ghost cells (aberrant keratinization) can be associated with varying amounts of dysplastic dentin.” A DGCT is typically a painless, asymptomatic enlargement in the oral and maxillofacial areas that is locally aggressive and can potentially recur [3-5].

Making up between 4% and 30% of all odontogenic tumors, odontomas are the most prevalent benign mixed odontogenic tumors. It is considered a hamartomatous lesion composed of dental tissues such as enamel, dentin, pulp, and cementum. According to WHO 2017 classification, odontomas are divided into complex odontomas and compound odontomas [4-6].

This article's goal is to report a rare DGCT case connected to a compound odontoma and an impacted molar in the maxillary posterior area.

## Case presentation

**Figure 1:**
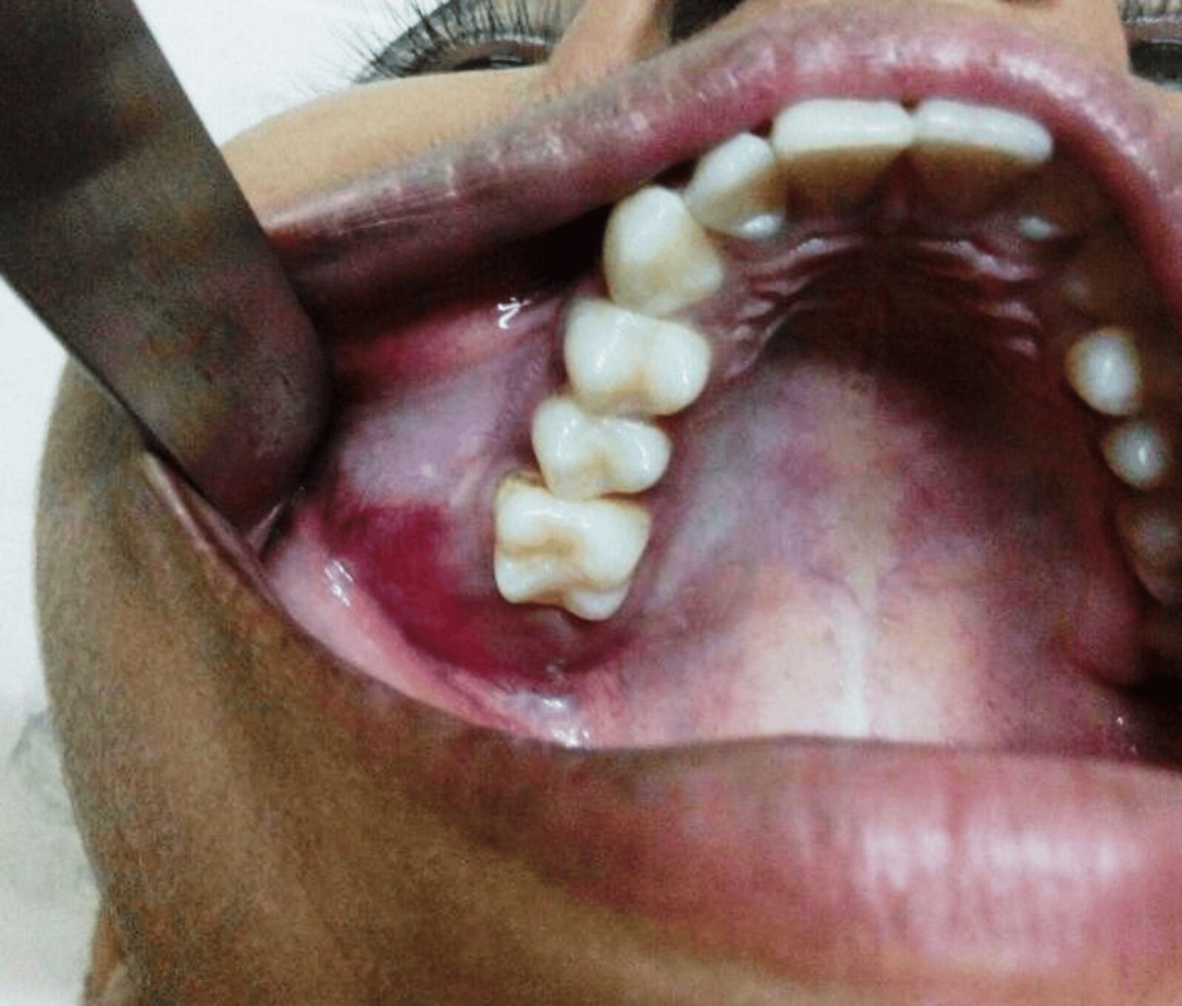
Intraoral swelling in the right upper back tooth region with obliteration of buccal vestibule

A 13-year-old female patient reported persistent swelling in her right cheek region for six months, accompanied by slight facial asymmetry, without any pain or discharge, despite non-contributory medical, dental, or family history. Intraoral examination revealed swelling of 5x4 cm, extending from permanent maxillary right first premolar (tooth number 14) to maxillary tuberosity, obliterating the buccal vestibule. The surface was normal with no secondary changes (Figure 1).

With the above clinical history, the clinical differential diagnosis of ameloblastoma, calcifying epithelial odontogenic tumor (CEOT), adenomatoid odontogenic tumor (AOT), odontogenic myxoma, and COC was given.

**Figure 2:**
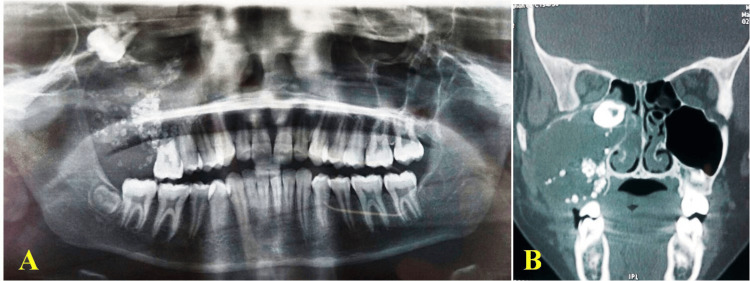
(A) Orthopantamogram with mixed radiolucency and radioopacity; (B) cone-beam computed tomography with obliteration of right maxillary sinus, impacted molar, and numerous radiopaque masses

A panoramic radiograph (Figure 2A) and cone beam computed tomography (CBCT) (Figure 2B) showed mixed radiolucency in the right maxilla (5 x 5 cm), sinus obliteration, impacted permanent maxillary right second molar (17) below the orbital fossa, numerous radiopaque masses, and root resorption of permanent maxillary right first molar (16). Radiographic differential diagnosis includes COC, CEOT, AOT with calcification, ameloblastic fibro-odontoma, and complex odontoma.

Fine needle aspiration revealed turbid brown fluid, and hematoxylin and eosin (H&E)-stained cytology revealed degenerating epithelial cells with prominent cytoplasm and foamy histiocytes, indicating a degenerating epithelial cyst.

**Figure 3:**
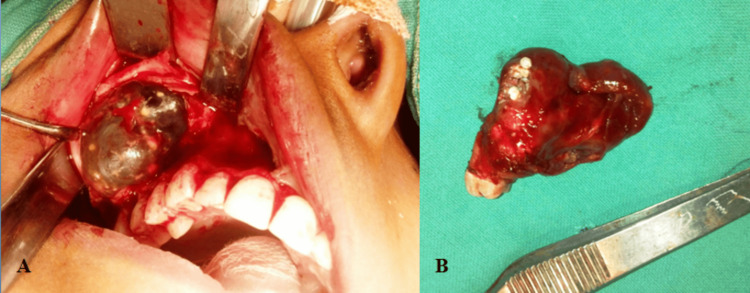
(A) Intraoperative picture of surgical excision; (B) excised specimen

The lesion was completely enucleated (Figure 3A), and the tooth associated with the lesion was also extracted and submitted for histopathology (Figure 3B). 

**Figure 4:**
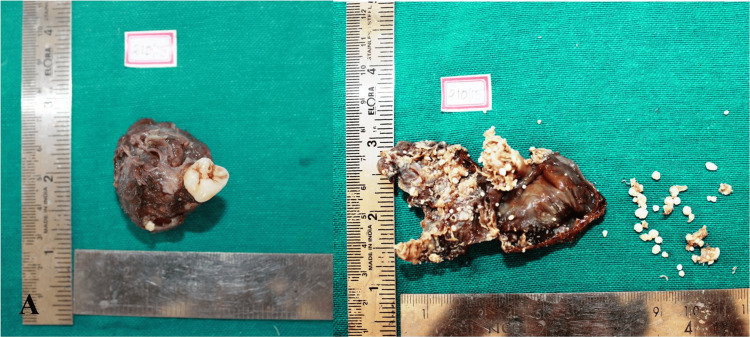
(A) Excised specimen after fixation with 10% formalin; (B) specimen after grossing shows multiple miniature tooth-like structures

On grossing, the lesion appeared cystic with an associated tooth (Figure 4A). The lesion was gritty to cut, with multiple miniature tooth-like structures attached to the cystic lining (Figure 4B).

**Figure 5:**
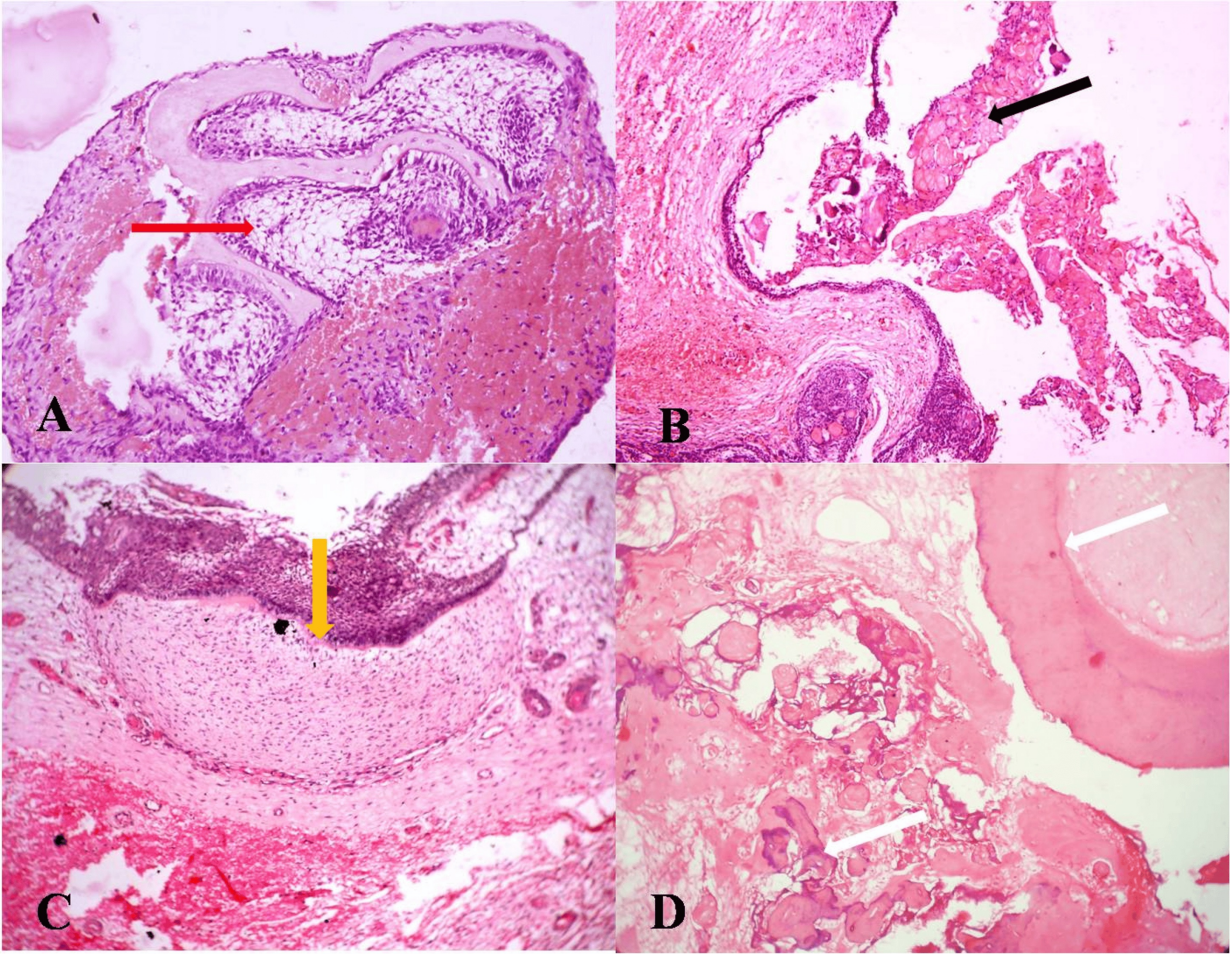
H&E-stained sections ameloblastoma-like areas with dysplastic dentin ((A) X200 magnification, red arrow), odontogenic cystic lining epithelium with ghost cells ((B) X100 magnification, black arrow), ameloblastoma-like epithelium with underlying primitive mesenchyme resembling dental papilla. ((C) X200 magnification, yellow arrow), decalcified section showing dysplastic dentin pulp and areas of cementum ((D) X200 magnification, white arrow)

The H&E-stained soft tissue section revealed a proliferating odontogenic cystic epithelial lining comprising basal columnar cells with a hyperchromatic nucleus showing reversal of polarity, resembling ameloblasts, with superficial polygonal cells appearing like stellate reticulum (Figure 5A). Numerous epithelium areas showed uniform eosinophilic masses with clear-out line-seeking ghost cells (Figure 5B). Connective tissue was biphasic, with few mature areas and focal areas of primitive mesenchyme reminiscent of dental papilla with dysplastic dentin (Figure 5C). The H&E-stained decalcified section revealed connective tissue cores resembling pulp tissue surrounded by dysplastic dentin in the cross- and longitudinal sections. Hematoxophilic areas resembling cementum were seen (Figure 5D). 

**Figure 6:**
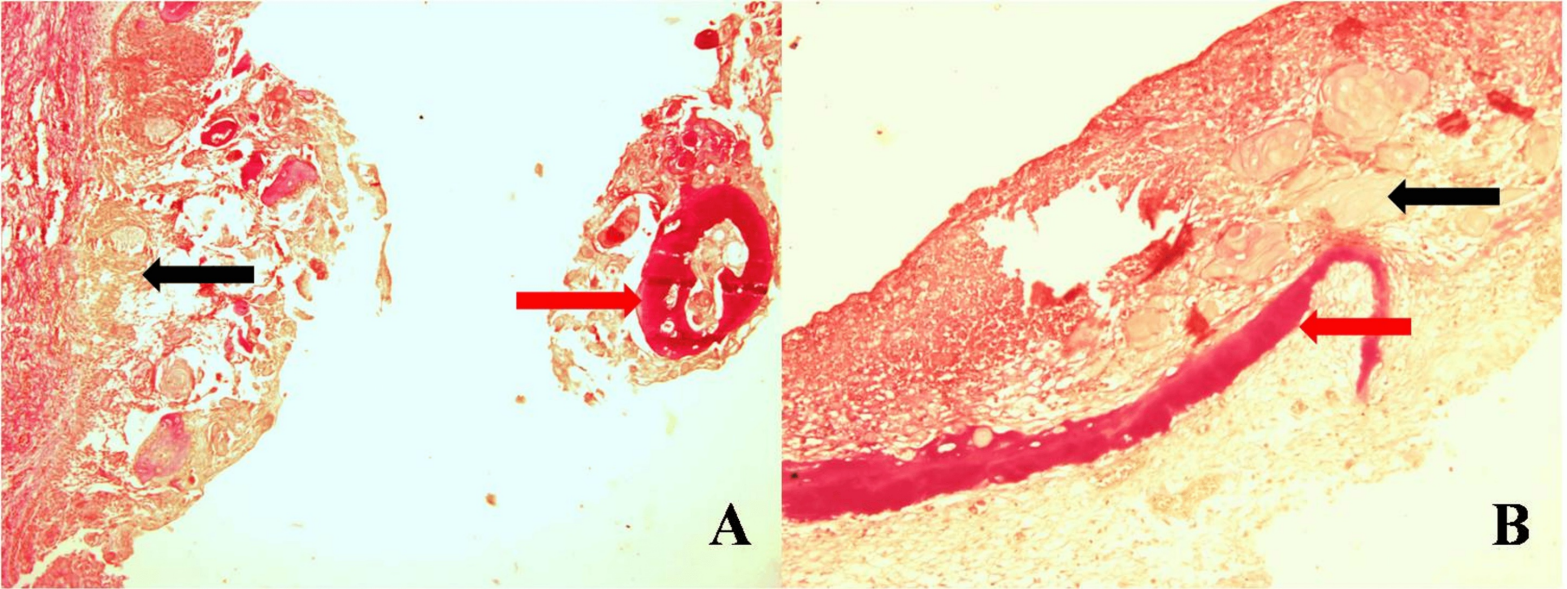
Van Gieson staining ((A) X100 magnification) and ((B) X200 magnification) odontogenic epithelium and ghost cells in yellow color (black arrow) and dentinoid material in pink color (red arrow)

Van Gieson staining showed odontogenic epithelium and ghost cells in yellow color and dentinoid material in pink color (Figure 6).

**Figure 7:**
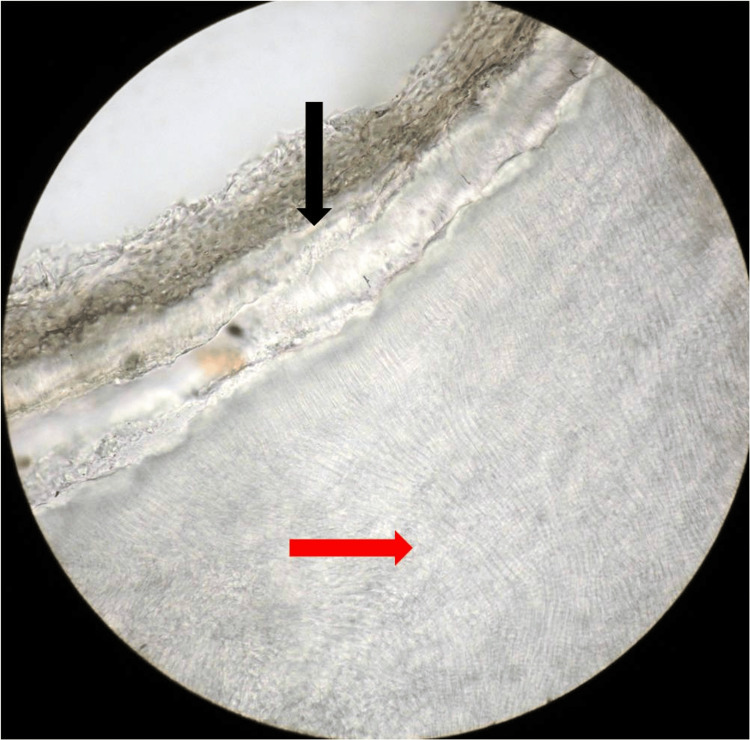
Ground section (X200 magnification) tubular dentin-like areas (red arrow) and cementum-like areas (black arrow)

The ground section revealed most tubular dentin-like areas, agglomerates of a tubular dentin-like at focal areas, and cementum-like areas (Figure 7). A DGCT with compound odontoma was the final diagnosis made based on clinical and histopathologic findings.

## Discussion

A DGCT is an exceptionally rare benign mixed epithelial-mesenchymal odontogenic tumor and constitutes between 0.2% and 0.5% of all odontogenic tumors. [1,2]. All age groups are susceptible to DGCT, while middle-aged to elderly people have higher frequency cases [5, 7]. In the literature, male predominance has been mentioned [7, 8]. A DGCT is a more often painless, slow-growing mass affecting the mandible than the maxilla, with a predilection for the posterior region [7]. There have been more reports of central or intraosseous patterns than peripheral or extraosseous patterns. [7,8]. The swelling was seen in the right maxillary region in our patient with gross facial asymmetry without any pain. 

When viewed radiographically, a DGCT is visible as a distinct, multilocular, radiolucent lesion with varying opacities that could be connected to impacted teeth. It has seldom been discovered to be connected to odontomas. In certain instances, root resorption and decay can be observed [9, 10]. In this instance, we observed impacted teeth surrounded by a radiolucent cystic cavity with small radiopaque masses and root resorption. Ameloblastic fibroadenomas and calcifying epithelial odontogenic tumors are two benign radiolucent lesions that are incorporated into the differential diagnosis from a clinical as well as radiographic standpoint. To our knowledge, only five cases of a DGCT associated with an odontoma have been reported in the literature [5-6, 11-13].

Histologically, dysplastic dentin and ghost cells are more frequently seen in fibrous tissue with odontogenic epithelial islands resembling ameloblastomas. It is thought that the DGCT epithelium is locally invasive. A conclusive diagnosis of DGCT will be possible with the occurrence of dentinoid growth and ghost cells [14]. In this case, we found a great deal of dentinoid material, ghost cells going through calcification, and ameloblastic nests and islands inside the stroma of connective tissue.

Odontomas and DGCTs may grow more quickly if the Wnt/beta (β)-catenin signaling chain is abnormally activated. Ghost cells are present in both tumors, recommending that the Wnt/β-catenin signaling chain may be involved in their development [15]. In particular, it has been noted that DGCTs have an elevated expression of the CTNNB1 gene, which codes for β-catenin, a crucial element of the Wnt/β-catenin pathway [16, 17]. These results imply that, as in this instance, anomalies in the Wnt/β-catenin pathway may cause odontoma and DGCT to arise together.

These genetic investigations might suggest that similar gene mutations or activations that happen at different times and places result in DGCT and odontoma being phenotypically distinct diseases.

Because of the destructive behavior and high recurrence rate observed in DGCT, wide surgical resection with safe margins has been recommended. Previous treatment with curettage and/or local enucleation has been associated with higher recurrence rates in DGCT. Therefore, simple surgical excision with additional bone curettage appears to be an effective approach [7].

There have been reports in the literature of the malignant transition of a DGCT to an odontogenic ghost cell carcinoma. Recurrence rates may reach 71%. While no recurrences have been documented in cases involving extraosseous tissue, recurrences usually happen five to eight years after the first therapy [18].

Taking into consideration the patient's age, the extent of the lesion, and the approximation of the lesion to the important structures such as the orbit, simple surgical excision with curettage and bone grafting were done. No recurrence was observed six months postoperatively.

## Conclusions

A DGCT is a very rare and locally aggressive lesion, and its occurrence with odontoma makes it unique and necessitates the diagnosis and treatment of a multidisciplinary team. The preferred course of treatment is wide surgical excision with safe margins, and long-term monitoring is required to determine the likelihood of recurrence. The likelihood of a malignant transformation in DGCT is quite low, making the prognosis generally favorable. Additional investigation is required to know the etiology of the co-occurrence of a DGCT with an odontoma.
